# Knowledge of final year undergraduate nursing students about HIV and AIDS in Eswatini

**DOI:** 10.4102/safp.v64i1.5527

**Published:** 2022-09-07

**Authors:** Makhosazana C. Dlamini, Ellen M. Thobakgale, Indiran Govender

**Affiliations:** 1 Department of Nursing, Faculty of Health Care Sciences, Sefako Makgatho Health Sciences University, Pretoria, South Africa; 2 Department Family Medicine and Primary Health Care, Faculty of Health Sciences, Sefako Makgatho Health Sciences University, Pretoria, South Africa

**Keywords:** undergraduate nursing students, knowledge, HIV/AIDS, people living with HIV and AIDS, Eswatini, nursing schools

## Abstract

**Background:**

Human immunodeficiency virus (HIV) and acquired immune deficiency syndrome (AIDS) are overwhelming health issues globally. They have caused many devastating and draining health issues, which have escalated a critical need for a well-trained and sustainable healthcare workforce in order to meet the needs of people living with HIV and AIDS (PLWHA). Health science students are the future healthcare providers who will implement proper preventive measures, as well as health educational and promotional sessions to promote information and knowledge among the public regarding HIV and AIDS in Eswatini.

**Methods:**

A quantitative cross-sectional study was conducted on 140 final-year undergraduate nursing students in three nursing universities in Eswatini. A questionnaire adapted from Othman and Ali in Malaysia with closed-ended questions was modified and used to collect data. The questionnaire consisted of questions on the virus structure, transmission, prevention and management of HIV and AIDS. Statistical Package for the Social Sciences (SPSS) version 20 was utilised to analyse the data.

**Results:**

The level of knowledge about HIV and AIDS was high, as evidenced by a mean score and standard deviation of (91.02 ± 5.00). However, there were low scores on questions related to the transmission of the disease.

**Conclusion:**

Across all three universities in Eswatini, there were good nursing education programmes on HIV and AIDS, evidenced by the high knowledge level about HIV and AIDS. However, there are still some knowledge gaps on HIV and AIDS transmission and management that need to be attended to.

**Contribution:**

This study contributed by providing knowledge of undergraduate nursing students’ HIV and AIDS training and management of PLWHA.

## Introduction

Human immunodeficiency virus (HIV) and acquired immune deficiency syndrome (AIDS) are among the devastating health issues in Eswatini.^1,2^ The United Nations programme on HIV and AIDS (UNAIDS) reported 220 000 people living with HIV and/or AIDS (PLWHA) in Eswatini.^3^ The rise in HIV and AIDS puts Eswatini at risk and vulnerable to new HIV infections and AIDS-related deaths.^3^ The risks also include the orphaned children who are vulnerable. The United States Agency for International Development (USAID) estimated 130 000 orphans and vulnerable children in Eswatini,^4^ while UNAIDS reported that about 67% of the PLWHA are on treatment.^3^

With the ever-increasing numbers of HIV and AIDS patients, doctors and nurses taking care of these patients should have adequate knowledge of the disease, and their attitude and behaviour should be appropriate.^5^ The knowledge of healthcare professionals in relation to HIV and AIDS is important in increasing their confidence to provide quality care to HIV-positive patients. The higher the knowledge level regarding HIV and AIDS, the higher is the willingness to care for AIDS patients among nursing students.^6^

Furthermore, UNAIDS reported 31% HIV and AIDS-related deaths^3^ that could be because of lack of knowledge. With such a burden, hospitals and clinics always have high numbers of patients with HIV and AIDS-related problems.^3^ To curb the pandemic in Eswatini, USAID suggested that a large number of health personnel be equipped with knowledge during their training.^4^ In this case, health personnel includes nurses and undergraduate nursing students who provide care to PLWHA.^4^

The fast-growing increase in disease prevalence has raised emotions and fears among healthcare providers, including undergraduate nursing students. If left unattended, these fears may produce a barrier to successful education efforts about the disease and may result in detrimental outcomes. Although Eswatini has made great progress to expand access to HIV testing and counselling (HTC) and implementing nursing education programmes on HIV and AIDS, additional and advanced HIV and AIDS-related information and knowledge among healthcare providers, inclusive of undergraduate nursing students, are urgently and continually needed.

In India, Kalyanshetti and Nikam^7^ reported an existing knowledge gap about HIV and AIDS among undergraduate nursing students. The existing gaps were observed on transmission routes and the general appearance of HIV-infected people.^5^ Knowledge, in this case, could influence the quality and level of care of PLWHA.^8^ This study sought to determine the knowledge of final-year undergraduate nursing students about HIV and AIDS in Eswatini.

## Methods

A quantitative, cross-sectional study was conducted to describe and provide information on the knowledge of final-year undergraduate nursing students in Eswatini universities. The study population comprised 170 undergraduate nursing students, both male and female, in their final-year level at the nursing universities in Eswatini. A nonprobability convenience sampling method was used. The questionnaires were anonymous. Data were collected during the academic year 2018–2019. Students were approached before their lectures. The study was explained to them and permission and consent were sought. The researchers could only collect data on specific dates, as the institution gave limited dates on which to collect data so as not to interrupt teaching and assessments. If they consented to participate, they filled in the questionnaires in their own time. The completed questionnaires were placed in a box at one of the offices. The sample size of 115 respondents was estimated by assuming the effect size of 0.15,^9^ type 1 error alpha at 0.05, two-tailed, with statistical power of 0.8 using G power version 3.15.^10^ Allowing for a 20% attrition rate, researchers hoped to recruit 150 respondents (Table 1). However, the final sample of respondents who consented was 140, which was higher than the statistically calculated representative sample size of 115. The three universities in the study were Eswatini Medical Christian University (EMCU), Southern Africa Nazarene University (SANU), University of Eswatini (UNESWA).

**TABLE 1:** Sample distribution table.

Universities	Estimated number of graduates	Sample size
EMCU	50	40
UNESWA	30	25
SANU	90	85

EMCU, Eswatini Medical Christian University; SANU, Southern Africa Nazarene University; UNESWA, University of Eswatini.

A restructured adapted questionnaire was used to collect data. The questionnaire used to restructure the data collection tool of this study was adapted from Othman and Ali^11^ in a study in Malaysia. The questionnaire comprised two sections: section 1 gathered demographic characteristics and section 2 concerned the HIV and AIDS knowledge of undergraduate nursing students. The Cronbach’s alpha coefficient of the adopted tool was calculated to assess its internal consistency; it was α = 0.74, which was considered acceptable. The questionnaire was reported to be reliable.^12^ The questionnaire was adapted to include knowledge questions and the local setting of the study.

Descriptive statistics were used to analyse the data. The Statistical Package for the Social Sciences (SPSS) version 20 was utilised to analyse and to calculate percentages, means and standard deviation (s.d.). The scores were summarised and compared, as suggested by De Vos et al.^13^ A statistical difference of less than a 0.05 level was considered statistically significant. Demographic variables were analysed using mean s.d. and frequency. Demographic characteristics of age versus knowledge were analysed using analysis of variance (ANOVA), while gender versus knowledge was analysed using the independent *t*-test, and university versus knowledge was analysed using ANOVA. There were 34 knowledge questions, each scored on a scale of 3 (unlikely = 0, false = 1, possible = 2, true = 3), to give a maximum total score of 102.

### Ethical considerations

Ethical clearance to conduct the study was obtained from Sefako Makgatho Health Sciences University Research and Ethics Committee (ref. no. SMUREC/H/224/2018:PG). Permission was sought from the National Health Research Review Board (ref. no. NHRRB REC 210408-003) of Eswatini. Furthermore, permission was sought from the management in the three universities, the Nursing Science Department and undergraduate nursing students. The respondents were informed of the aim of the study, and written consent was obtained.

## Results

There were 140 respondents, and there was statistically significant representation from all three universities. The mean age of the respondents was 24.8 ± 3.3 (mean ± s.d.), and this mean age also shows that a majority of undergraduate nursing students being trained are still in their active years.

Female undergraduate nursing students dominated male undergraduate nursing students with just a small difference. Of the 140 nursing students, 25.0% were from EMCU, 56.6% from SANU and 16.4% from UNESWA. Swazi origin was 99.3%, as compared with 0.7% of non-Swazi origin. Ninety percent of the undergraduate nursing students were single, and only 10.0% were married. Demographic characteristics of the sample are shown in Table 2.

**TABLE 2:** Demographic characteristics of students (*n* = 140).

Variables	*N*	%	Mean ± s.d.
**Age**			24.8 ± 3.3
20–25	103	73.6	
26–30	28	20.0	
≥ 31	9	6.4	
**University**			
EMCU	35	25.0	
SANU	82	56.6	
UNESWA	23	16.4	
**Gender**			
Male	59	42.1	
Female	81	57.9	
**Ethnic group**			
Swazi	139	99.3	
Non-Swazi	1	0.7	
**Marital status**			
Single	126	90.0	
Married	14	10.0	

EMCU, Eswatini Medical Christian University; SANU, Southern Africa Nazarene University; UNESWA, University of Eswatini; s.d., standard deviation.

From Table 3, the knowledge total item score was high, with a mean score and s.d. of (91.02 ± 5.00). Results indicated that all students (3.00 ± 0.00) knew that AIDS was caused by a virus that attacks the body’s immune systems. Many of the students (2.72 ± 0.62) agreed that a person can be infected with HIV for five years or more without getting AIDS. Students also displayed a good level of knowledge regarding PMTCT, with a mean score 2.99 ± 0.85; however, some of them did not know if PMTCT was an initiative for all women or if it excludes single pregnant women (2.54 ± 0.85). Again, the mean score of 2.27 ± 1.07 highlights a low knowledge about the window period.

**TABLE 3:** Students’ knowledge descriptive statistics (*n* = 140).

Variable	Knowledge Mean ± s.d.	Rank
AIDS is not the cause of HIV.	2.80 ± 0.61	18
A person cannot get HIV by sharing a glass of water with someone who has HIV.	2.79 ± 0.73	19
There is no cure for AIDS.	2.81 ± 0.57	17
HIV and AIDS are not the same thing.	2.91 ± 0.35	8
A pregnant woman with HIV can give the virus to her unborn baby.	2.53 ± 0.84	28
Pulling out the penis before climax does not protect a woman from getting HIV during sex.	2.95 ± 0.33	5
A woman can get HIV if she has anal sex with a man.	2.69 ± 0.59	21
Showering or washing one’s genitals or private parts after sex will not prevent a person from getting HIV.	2.84 ± 0.63	15
Not all pregnant women infected with HIV will have babies born with AIDS.	2.85 ± 0.52	14
Couples in polygamous families are at high risk of getting HIV	2.88 ± 0.54	11
Using a latex condom or rubber can lower a person’s chance of getting HIV.	2.84 ± 0.48	16
A person who has been infected with HIV does not quickly show serious signs of infection.	2.52 ± 0.96	29
A person can be infected with HIV for 5 years or more without getting AIDS.	2.72 ± 0.62	20
HIV can be transmitted through oral sex.	2.59 ± 0.94	24
A vaccine against HIV has not been found yet.	2.58 ± 0.89	25
No drugs have been made for the treatment of AIDS.	2.23 ± 0.94	32
A woman is not safe from HIV if she has sex during her period.	2.87 ± 0.53	12
People who are HIV-positive look and feel healthy.	2.43 ± 0.68	30
Female condoms help decrease a woman’s chance of getting HIV.	2.87 ± 0.41	13
HIV can pass to someone during sexual intercourse.	2.96 ± 0.19	4
AIDS is caused by a virus that attacks the body’s immune system.	3.00 ± 0.00	1
Proper or good nutrition is essential in the management of HIV and AIDS patients.	2.89 ± 0.32	10
HIV reduces the body’s natural defence mechanisms against infections.	2.96 ± 0.28	3
HIV-positive mothers can transmit the virus to their babies, not only during delivery.	2.56 ± 0.78	26
All pregnant women are advised to screen for HIV in the early stages of pregnancy.	2.99 ± 0.85	2
PMTCT is not an initiative for single pregnant women.	2.54 ± 0.85	27
Condoms are the only effective means of reducing HIV transmission.	1.57 ± 0.87	33
A person will get HIV even if she or he is taking antibiotics.	2.90 ± 0.42	9
Having sex with more than one partner can increase a person’s chance of being infected with HIV.	2.94 ± 0.32	6
Taking a test for HIV 1 week after having sex will not tell a person if she or he has HIV.	2.27 ± 1.07	31
A person can get HIV through contact with saliva, tears, sweat or urine.	1.56 ± 1.06	34
A person can get HIV from a woman’s vaginal secretions.	2.65 ± 0.66	22
If a person tests positive for HIV, the test site will not tell all of his or her partners.	2.59 ± 0.96	23
Athletes who share needles when using steroids can get HIV from the needles.	2.93 ± 0.37	7

**Knowledge total item score**	**91.02 ± 5.00**	**-**

PMTCT, prevention of mother-to-child transmission; s.d., standard deviation; HIV, human immunodeficiency virus; AIDS, acquired immune deficiency syndrome.

The mean score showing understanding of modes of transmission of HIV, which included ‘having multiple partners’, ‘sharing needles’ and ‘pulling out penis before climax does not protect the women from getting HIV’, was (2.94 ± 0.32), (2.93 ± 0.37) and (2.95 ± 0.33), respectively. However, transmission through other sex types got lower scores, with anal sex 2.69 ± 0.59 and oral sex 2.59 ± 0.94. The majority of the undergraduate nursing students lacked understanding about whether a person could get HIV through contact with saliva, tears, sweat or urine, with a mean score of (1.56 ± 1.06).

In addition, the mean score of 2.81 ± 0.57 demonstrates that a majority of the undergraduate nursing students knew that there was no cure for HIV or AIDS. Yet 2.58 ± 0.89 agreed that a vaccine against HIV has also not been found, whereas there was a decrease in knowledge level regarding the availability of drugs used in the management of HIV and AIDS that is shown by a low score of 2.23 ± 0.94. Many of the students did not know that condoms are an effective means for reducing HIV transmission. This question had a low mean score of 1.57 ± 0.87. In summary, the undergraduate nursing students’ knowledge regarding mother-to-child transmission of HIV infection was 97.9%; 95.7% of them agreed that having multiple partners and sharing needles increases ones’ chances of being infected with HIV; 87.1% knew that HIV and AIDS were not curable; and 77.9% knew that a vaccine against HIV has not been found.

In Table 4, the study evaluated possible demographic factors influencing students’ knowledge. Results showed no statistical significance between age and knowledge (*r* = 0.96, *p* = 0.91) and no statistical significance in gender against knowledge (*r* = −1.07, *p* = 0.29). However, there was a strong statistical significance association discovered between the age groups 20–25 and 31 years and above (*t* = 3.22, *p* = 0.04).

**TABLE 4:** Factors influencing students’ knowledge on human immunodeficiency virus and acquired immune deficiency syndrome (*n* = 140).

Variable	*N*	Knowledge *post hoc*		
		Mean ± s.d.	*F/t/r*	*p* *
**Age**			0.96	0.91
20–25	103	90.92 ± 5.15		
26–30	28	91.39 ± 4.64		
≥ 31	9	91.00 ± 3.80		
**Gender**			−1.07	0.29
Male	59	90.49 ± 4.73		
Female	81	91.41 ± 5.18		
**University**			0.67	0.52
EMCU	35	90.23 ± 5.90		
SANU	82	91.39 ± 4.54		
UNESWA	23	90.91 ± 5.16		

EMCU, Eswatini Medical Christian University; SANU, Southern Africa Nazarene University; UNESWA, University of Eswatini; s.d., standard deviation.

* , Correlation was set to be significant at the 0.01 level (two-tailed).

## Discussion

Determining undergraduate nursing students’ knowledge on HIV and AIDS is a priority to improve professional educational programmes. The present study demonstrated that undergraduate nursing students had a high level of knowledge. This is consistent with previous reports where students showed a high level of knowledge regarding HIV and AIDS, their causes, pathophysiology and management.^14^

Inconsistent findings have been presented in other countries, where the research showed that undergraduate nursing students’ knowledge was relatively low regarding HIV and AIDS worldwide.^15^ The knowledge of undergraduate nursing students is reported to have been very high in other studies^16,17^ and moderate or inadequate in some studies.^18,19^

While the cure for HIV and AIDS has still not been found, the struggle to deal with the condition remains; thus, it is essential for undergraduate nursing students and other healthcare students to have detailed knowledge about HIV and AIDS. In this study, all the undergraduate nursing students agreed that a virus that attacks the body’s immune system causes AIDS, and this is consistent with previous studies.^20,21^

In this study, the undergraduate nursing students’ knowledge regarding mother-to-child transmission of HIV infection was high (97.9%), showing positive knowledge that all pregnant women are advised to screen for HIV in the early stages of pregnancy. This conflicts with the results of a study that was conducted among Faculty of Health Sciences nursing students at a private university in Malaysia. The results revealed that they lacked knowledge regarding mother-to-child transmission, which was worrying, according to Amin and Awang.^22^ However, some of the undergraduate nursing students did not know that PMTCT was an initiative for all women, even single pregnant women. Their lack of knowledge is seen as the gap that needs to be filled.^23^ Their lack of knowledge could be related to a lack of exposure to PMTCT practice because of very limited spaces in clinical areas.

Similarly, the undergraduate nursing students continued to demonstrate good knowledge in the context of HIV transmission. A total of 95.7% of them agreed that having multiple partners and sharing needles increases ones’ chances of being infected with HIV.

This is in agreement with studies conducted previously, where nursing students scored over 90% on items related to transmission.^19,22,24^ Although some studies may demonstrate such good knowledge, in other studies, students stated that HIV cannot be transmitted by use of unsterilised utensils, with some highlighting that it can be passed via insect bites.^18^

Furthermore, the majority (87.1%) of students knew that HIV and AIDS were not curable and that a vaccine against HIV has not been found (77.9%). In Kok et al.,^14^ the undergraduate nursing students demonstrated the same knowledge level when they agreed that HIV cannot be prevented by vaccination. Meanwhile, other studies indicated that students’ knowledge was inadequate in this regard, as most students were not aware that there was no cure or vaccine for HIV.^25,26^ Such misinformation on knowledge regarding the available management methods or treatment of HIV and AIDS calls for the need for early exposure and monitoring of the undergraduate nursing students,^18^ because it indicates gaps in the knowledge on the management of HIV and AIDS.

As prevention is regarded as the only solution for HIV and AIDS reduction, the undergraduate nursing students in this study demonstrated good knowledge on the methods for preventing HIV and AIDS. However, they were confident that condoms are the only, best and real method for the prevention of HIV and AIDS. Kok et al.^14^ reiterated that the undergraduate nursing students had a great deal of confidence in condom usage as the only effective way of preventing HIV and AIDS transmission.^12^ Half of the undergraduate nursing students from Benin University in Nigeria lacked knowledge on the mode of transmission. Esewe et al.^20^ reported that they strongly agreed that patients living with HIV and AIDS should not be admitted into the same ward with other patients, with the aim of preventing spread.^20^

## Conclusion

For healthcare professionals, inclusive of undergraduate nursing students, knowledge on HIV and AIDS is necessary when providing quality care to PLWHA. In general, undergraduate nursing students exhibited good knowledge on HIV and AIDS, especially regarding the nature of HIV and AIDS, the mode of transmission and management. However, there were a few misconceptions observed on other aspects of knowledge, which need to be addressed, including knowledge on transmission and management of the disease. Some nursing students believed that a person could get HIV through contact with saliva, tears, sweat and urine, and that is a misconception.

The researchers, through the results of the study especially on the knowledge of the nursing students, have appreciated the steps taken by the Eswatini Nursing Council (ENC) on improving HIV and AIDS knowledge among nursing students. The ENC and the nursing schools have made good strides and need to identify gaps and make amendments in order to reach a highly desirable level of knowledge across all nursing students, which will further result in highly knowledgeable nurses. The country also has a huge responsibility to conduct more research studies related to HIV and AIDS among nursing students and other healthcare professionals.

## Limitations of the study

Although the statistically calculated significant sample size was 150, only 140 students consented to participate in the study. This may have impacted the results of the study. The convenient sampling method employed may have contributed to selection bias. This sampling method was employed because of time constraints and we wanted to collect data while the students were in a large group attending lectures. At most times the students are dispersed in small groups doing clinical learning. The results of this study may only be generalisable to these three training sites in this country.
